# A Complete Encyclopædia of State Medicine

**Published:** 1843-01

**Authors:** 


### PART SECOND.

### Bibliographical Notices.

---

ART. I.—*Ausführliche Encyklopädie der gesammten Staatsarzneikunde.* Von GEORG FRIEDERICH MOST, Doctor der Philosophie, Medicin und Chirurgie.—*Leipzig,* 1838-9-40. Zwei Bände. 8vo, pp. 1132, 1190.

*A Complete Encyclopædia of State Medicine.* By Dr. GEORGE FRED. MOST. In Two Volumes.—*Leipzig,* 1838-9-40.

IN a former number of the Review (No. XIV, April 1839, p. 530,) we noticed the first appearance of the Encyclopædia of Dr. F. Most. We at that time expressed the opinion that this was not a convenient form for conveying practical information on the science. That opinion is confirmed by the examination of the two enormous volumes in which the work is now completed. The title strictly implies a Dictionary of State Medicine, but the articles refer to almost every conceivable subject belonging to the physical and moral sciences. Thus articles on every branch of medicine and surgery are mixed up with essays on metaphysics, mesmerism, homœopathy, and quackery. There is an article also on temperance societies (Mässigkeits-gesellschaften), in which the political and medical consequences likely to result from the diffusion of temperance principles are discussed. In short, this work would be better entitled an Encyclopædia of general Knowledge; for in most of the essays the original object of the editor seems to have been entirely abandoned. We are not surprised that an attempt of this kind should have failed: it is another illustration of the old Latin proverb, " ne quid nimis." Had Dr. Most confined the work to subjects strictly appertaining to medical jurisprudence and police, it would have been reduced to one third of its present unwieldy size, and might have made a volume of good practical information.

As we might have anticipated it has been found necessary to add many omitted articles, in the shape of an appendix. This appendix is not yet completed, and, judging from appearances, it is likely of itself to form a small encyclopædia.

It is only fair to the editor to observe that many of the essays on medical jurisprudence, as well as those on medical police, are very creditably written. They are, for the most part, compilations from well-known authorities. Thus the subject of toxicology is almost entirely extracted from the works of Orfila, Christison, and others. The article on arsenic contains no reference to Marsh's test, or Orfila's late discoveries, not even in the appendix! In general, however, the subject of toxicology is very well treated. The essay on infanticide, which is rich in historical is deficient in practical information. There are one or two excellent articles on the general rules to be pursued in the analysis of poisons, where the nature of the substance is unknown. This is a subject not commonly treated in works on toxicology, and we therefore refer to them those who may have access to the work. We do not make any extracts from this Encyclopædia in reference to medico-legal subjects, because by so doing we feel we should only be reproducing what has already appeared in this Journal, in reviewing the works of Orfila, Devergie, and others.

Dr. Most's work, although essentially written with a view to German practice and principles, contains information which will be useful to scientific men of all countries. We fear, however, from its size and plan, its circulation out of Germany will be limited. To an Englishman it would certainly be a matter of considerable difficulty, to know where to refer for the information which he might require.

